# Genetic Characterization of Plasmid-Borne *bla*_OXA-58_ in Distinct Acinetobacter Species

**DOI:** 10.1128/mSphere.00376-19

**Published:** 2019-10-16

**Authors:** Adriana P. Matos, Rodrigo Cayô, Luiz G. P. Almeida, Ana Paula Streling, Carolina S. Nodari, Willames M. B. S. Martins, Ana Clara Narciso, Rosa M. Silva, Ana T. R. Vasconcelos, Ana C. Gales

**Affiliations:** a Universidade Federal de São Paulo—UNIFESP, Laboratório Alerta, Disciplina de Infectologia, Departamento de Medicina, Escola Paulista de Medicina—EPM, São Paulo, São Paulo, Brazil; b Universidade Federal de São Paulo—UNIFESP, Laboratório de Imunologia e Bacteriologia—LIB, Setor de Biologia Molecular, Microbiologia e Imunologia—Departamento de Ciências Biológicas—CDB, Instituto de Ciências Ambientais, Químicas e Farmacêuticas—ICAQF, Diadema, São Paulo, Brazil; c National Laboratory for Scientific Computing—LNCC, Petrópolis, Rio de Janeiro, Brazil; d Universidade Federal de São Paulo—UNIFESP, Laboratório de Microbiologia Básica, Department of Microbiology, Immunology and Parasitology, Escola Paulista de Medicina—EPM, São Paulo, São Paulo, Brazil; Antimicrobial Development Specialists, LLC

**Keywords:** *Acinetobacter calcoaceticus*-*Acinetobacter baumannii* complex, Gram-negative bacilli, carbapenem resistance, carbapenem-hydrolyzing class D β-lactamase, nosocomial infection

## Abstract

We characterize by whole-plasmid-sequence (WPS) two-plasmid-borne *bla*_OXA-58_ obtained from Acinetobacter seifertii (Asp-1069) and A. baumannii (Acb-45063) clinical strains recovered 17 years apart from distinct Brazilian regions. Multilocus sequence type (MLST) analysis showed that the Asp-1069 and Acb-45063 strains belong to ST551 and ST15/CC15, respectively. WPS analysis demonstrated that *bla*_OXA-58_ was located in two distinct plasmids named pAs1069_a (24,672 bp/44 open reading frames [ORFs]) and pAb45063_b (19,808 bp/24 ORFs), which belong to the GR8/GR23 (*repAci23*) and GR4 (*repAci4*) incompatibility groups, respectively. The genetic environments surrounding *bla*_OXA-58_ revealed that it was flanked by two intact IS*Aba3* copies on pAb45063_b, which differed from pAs1069_a. In the latter, the upstream IS*Aba3* copy was truncated by insertion of IS*Aba825* element. Although Re27-specific recombination sites were found adjacent to IS*Aba3-bla*_OXA-58_-IS*Aba3* arrangement on pAb45063_b, such structures were absent on pAs1069_a. The conserved IS*Aba125*-*araC1*-*lysE* arrangement was disrupted by Tn*aphA6* harboring the aminoglycosides resistance gene *aphA6* on pAs1069_a, while an IS*26*-*bla*_TEM-1_-*aac(*3*)-IIa*-IS*26* genetic structure was found upstream from IS*Aba3-bla*_OXA-58_-IS*Aba3* on pAb45063_b. Other two plasmids, pAb45063_a (183,767 bp/209 ORFs) and pAs1069_b (13,129 bp/14 ORFs), were also found in the OXA-58-producing Acinetobacter species strains, harboring the *strA* and *strB* genes and the *sul2* gene, which confer resistance to streptomycin and sulfonamides, respectively. The plasmid-mediated virulence factors corresponding to genes *tonB*, *spl*, *glmM*, *ppa*, *sulP*, and *map* were found in both strains, as well distinct toxin-antitoxin system-encoding genes *stbD* and *relE* (pAs1069_a), *brnT* and *brnA* (pAb45063_b), and *xreE* (pAb45063_a). Although infrequently reported in Brazil, plasmid-borne *bla*_OXA-58_ showed a complex and diverse genetic backbone that confers stability in different Acinetobacter species that have been isolated from nosocomial settings over time.

**IMPORTANCE** Although the *bla*_OXA-58_ gene has been infrequently described in Brazil, contrasting with other bordering South American countries, we verified the maintenance of this resistance determinant over time among carbapenem-resistant Acinetobacter species isolates, not only in nosocomial settings but also in the environment. In addition, to the best of our knowledge, this is the first study to have used WPS analysis to evaluate the genetic surroundings of *bla*_OXA-58_ in Brazil. Moreover, the A. seifertii and A. baumannii clinical strains evaluated in this study were recovered 17 years apart in hospitals located in distinct Brazilian geographic regions.

## Abstract (precis)

Although the *bla*_OXA-58_ gene has been infrequently described in Brazil, contrasting with other bordering South American countries, we verified the maintenance of this resistance determinant over time among carbapenem-resistant Acinetobacter species isolates, not only in nosocomial settings but also in the environment. In addition, to the best of our knowledge, this is the first study to have used WPS analysis to evaluate the genetic surroundings of *bla*_OXA-58_ in Brazil. Moreover, the A. seifertii and A. baumannii clinical strains evaluated in this study were recovered 17 years apart in hospitals located in distinct Brazilian geographic regions.

## INTRODUCTION

Acinetobacter species are important pathogens frequently responsible for causing nosocomial infections, mainly in patients hospitalized at intensive care units (ICU) (1, 2). The spread of major carbapenem-resistant A. baumannii clones has been associated with the increasing frequency of the carbapenem resistance phenotype worldwide (1). The production of carbapenem-hydrolyzing class D β-lactamases (CHDLs) has been reported to be the main mechanism of carbapenem resistance (3). The spread of multidrug-resistant (MDR) A. baumannii sequence type 79 (ST79) isolates carrying *bla*_OXA-23_ and, more recently, *bla*_OXA-72_ has contributed to the high carbapenem resistance rates (85%) observed in Brazil (3). In contrast, *bla*_OXA-58_ has been rarely reported in this country, contrasting with the high frequency of this CHDL-encoding gene seen in neighboring countries, mainly in Argentina (4). To date, only six OXA-58-producing Acinetobacter species strains recovered from human (5–8) and environmental (9) sources have been reported in distinct Brazilian cities since the 90s (5–9). The diverse genetic structures surrounding *bla*_OXA-58_ documented worldwide (3, 10, 11) play a major role not only in the mobilization of this resistance determinant but also in driving its expression, decisively leading to the carbapenem resistance phenotype (3, 12).

The carriage of *bla*_OXA-58_ by distinct Acinetobacter species recovered over a long period of time—3 decades—suggests that this CHDL-encoding gene has been mobilized by horizontal gene transfer (HGT), although the hypothesis of clonal spread could not be completely discarded. In order to reveal the genetic environment of *bla*_OXA-58_ and the corresponding implications for the mobilization and maintenance of this resistance determinant over time, we molecularly characterize two distinct plasmids harboring *bla*_OXA-58_ obtained from A. seifertii and A. baumannii clinical strains recovered in the years 1993 and 2010, respectively, from distinct Brazilian geographic regions.

## RESULTS

**FIG 1:**
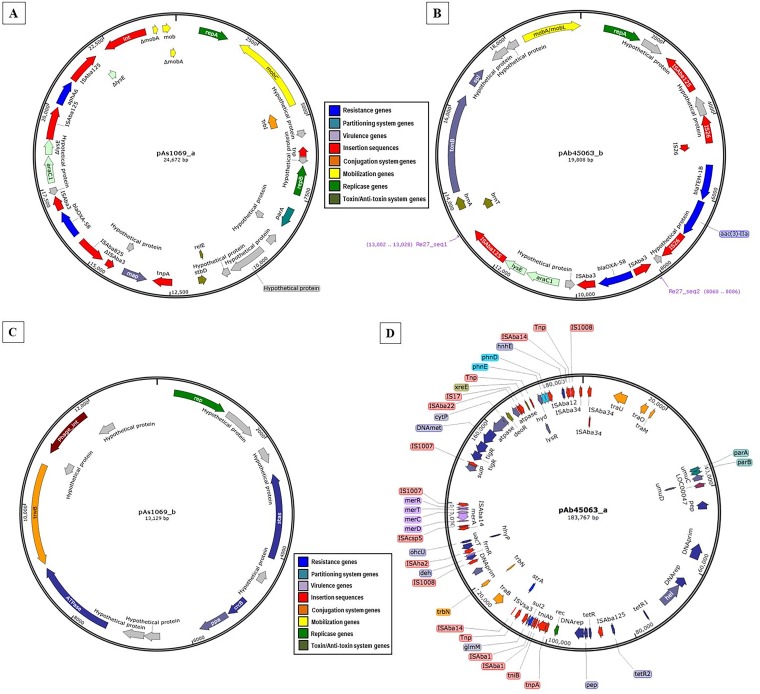
Schematic representation of circular plasmid maps found in A. seifertii Asp-1069 (A and C) and A. baumannii Acb-45063 (B and D) strains. Arrows designate transcription directions of genes and ORFs. Genes were grouped according to their predictive functions as indicated by the color coding key.

**FIG 2:**
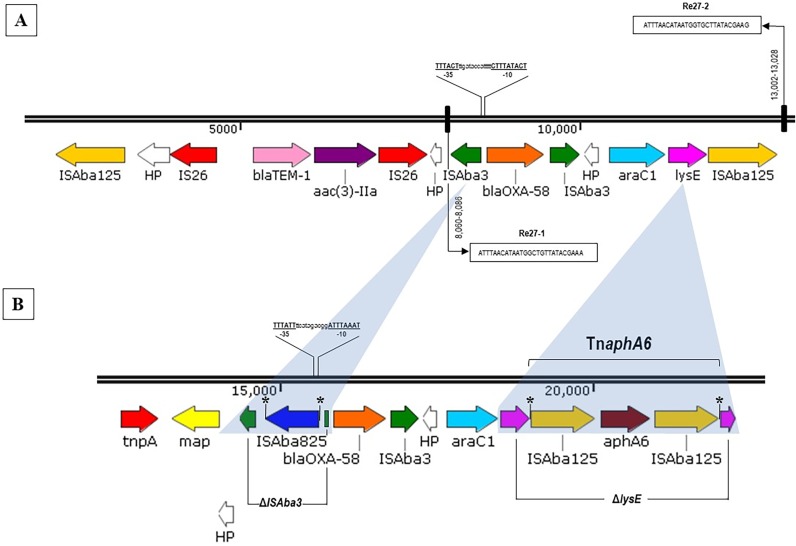
Genetic contexts surrounding *bla*_OXA-58_ found in plasmids pAb45063_b (A) and pAs1069_a (B). Genes and their transcriptional orientations are represented by horizontal arrows. Identical genes found in both genetic structures are represented with the same colors. Genes of no predicted functions (HP [hypothetical proteins]) are represented in white. The putative original promoters driving the expression of *bla*_OXA-58_ genes are highlighted. Direct repeat sequences are represented by an asterisk (*). Gene names preceded by an uppercase Greek delta (Δ) represent truncated genes, and the corresponding regions are shaded in gray. The Re27 regions are boxed. Promoter prediction for *bla*_OXA-58_ was performed using BPROM (SoftBerry).

Two distinct plasmids were obtained for each strain according to WPS analysis, and the plasmids were named pAs1069_a (24,672 bp/44 open reading frames [ORFs]) and pAs1069_b (13,129 bp/14 ORFs) for the A. seifertii Asp-1069 strain that belongs to ST551^IP^ (Institut Pasteur scheme). For A. baumannii Acb-45063 strain ST15/CC15^IP^, two plasmids were also detected and were named pAb45063_a (183,767 bp/209 ORFs) and pAb45063_b (19,808 bp/24 ORFs) (Table 1). Plasmids of similar sizes were observed by alkaline lysis gel analysis (data not shown), considering an accepted variation range of ±10%, as follows: ∼155-kb and ∼32-kb plasmids for strain Acb-45063, corresponding to pAb45063_a and pAb45063_b, respectively, and ∼32-kb and ∼21-kb plasmids for strain Asp-1069, corresponding to pAs1069_a and pAs1069_b, respectively. WPS analysis demonstrated that *bla*_OXA-58_ genes were carried by pAs1069_a/24,672-bp (Fig. 1A) and pAb45063_b/19,808-bp (Fig. 1B) plasmids, which belong to the A. baumannii replicon type group (AbGR) GR8/GR23 (*repAci23*) and GR4 (*repAci4*), respectively (Table 1). On pAb45063_b, *bla*_OXA-58_ was flanked by two intact copies of IS*Aba3* (Fig. 2A). In contrast, the genetic environment surrounding *bla*_OXA-58_ on pAs1069_a revealed an imperfect 5′ IS*Aba3* that was disrupted by an IS*Aba825* and an intact copy of IS*Aba3* downstream (Fig. 2B). Putative promoter regions of *bla*_OXA-58_ were predicted for pAb45063_b and for pAs1069_a, conferred by IS*Aba3* and by IS*Aba825*, respectively (Fig. 2A and B). IS*Aba825* generates a 4-bp duplication (AACT) upon transposition (Fig. 2B).

**TABLE 1:** Microbiological data and plasmid characterization of two OXA-58-producing Acinetobacter species clinical isolates recovered in Brazil^*a*^

Strain	Species	Yr of Isolation	Clinical specimen	MLST	MIC (μg/ml)												Plasmid	Size (bp)	ORF	G+C (%)	AbGR	Genetic marker(s)	
					CAZ	CRO	FEP	IPM	MEM	AMK	GEN	CIP	TIG	MIN	PMB	SUT						Resistance	Virulence
Asp-1069	A. seifertii	1993	Tracheal aspirate	ST551	256	128	128	32	32	256	512	1	0.5	0.25	4	>32	pAs1069_a	24,672	44	36.62	GR8	*aphA6*, *bla*_OXA-58_	*map*
																	pAs1069_b	13,129	14	35.65	NT		*ppa*

Acb-45063	A. baumannii	2010	Blood	ST15	256	512	64	32	16	8	512	64	1	0.25	0.06	>32	pAb45063_a	183,767	209	37.61	NT	*strA*, *strB*, *sul2*	*sulP*, *glmM*
																	pAb45063_b	19,808	24	38.51	GR4	*aac(*3*)-IIa*, *bla*_TEM-1B_, *bla*_OXA-58_	*tonB*, *sep*

a MLST, multilocus sequence typing; CAZ, ceftazidime; CRO, ceftriaxone; FEP, cefepime; IPM, imipenem; MEM, meropenem; AMK, amikacin; GEN, gentamicin; CIP, ciprofloxacin; TIG, tigecycline; MIN, minocycline; PMB, polymyxin B; SUT, trimethoprim-sulfamethoxazole; ORF, open reading frame; G+C, guanine-cytosine content; AbGR, Acinetobacter replicon type group; NT, nontypeable.

Two Re27 sequences were found adjacent to an IS*Aba3-bla*_OXA-58_-IS*Aba3* arrangement on pAb45063_b, with Re27-1 located upstream of 5′-IS*Aba3* and Re27-2 adjacent to IS*Aba125* located downstream of *araC1* and *lysE* genes (Fig. 2A), which coded for a threonine efflux protein and a transcriptional regulator, respectively. In contrast, we failed to identify Re27-like regions on pAs1069_a. In this plasmid, *lysE* was disrupted by Tn*aphA6*, which harbored aminoglycoside modifying enzyme (AME)-encoding gene *aphA6*. This AME confers resistance to gentamicin and amikacin. *aphA6* was flanked by two copies of IS*Aba125* in the same orientation (Fig. 2B), while Tn*aphA6* generates a 7-bp duplication (ATTCGCC) upon transposition (Fig. 2B). The production of aminoglycoside O-phosphotransferase AphA6 by A. seifertii Asp-1069 justified the high MICs for amikacin (256 μg/ml) and gentamicin (512 μg/ml) verified in such strain (Table 1). In addition, a genetic arrangement composed of two copies of IS*26* and the narrow-spectrum-β-lactamase-encoding gene *bla*_TEM-1_ and the AME-encoding gene *aac(*3*)-IIa* was also found upstream of IS*Aba3-bla*_OXA-58_-IS*Aba3* on pAb45063_b (Fig. 2A). AAC(3)-IIa confers a high level of resistance to gentamicin but not amikacin, justifying the phenotype observed for A. baumannii Acb-45063 (MICs of 512 and 8 μg/ml for gentamicin and amikacin, respectively; Table 1). Other two plasmids (pAb45063_a and pAs1069_b) were also detected in the OXA-58-producing Acinetobacter species strains evaluated in the present study (Fig. 1C and D). The 183,767-bp plasmid Ab45063_a carried the streptomycin resistance genes *strA* and *strB* and the sulfonamide resistance gene *sul2* (Fig. 1D), contrasting with the small plasmid pAs1069_b of 13,129 bp (Fig. 1C). Although the two OXA-58-producing Acinetobacter species strains showed similar profiles of susceptibility to β-lactams, A. baumannii Acb-45063 MICs were 0.06 μg/ml and 64 μg/ml for polymyxin B and ciprofloxacin, respectively, contrasting with those presented by A. seifertii Asp-1069 strains (MICs of 4 and 1 μg/ml for polymyxin B and ciprofloxacin, respectively) (Table 1).

Distinct virulence factors were observed in all four plasmids (Table 1) as follows: outer membrane protein-encoding gene *tonB*, septicolysin-encoding gene *spl*, phosphoglucosamine mutase-encoding gene *glmM*, inorganic pyrophosphatase-encoding gene *ppa*, sulfate permease-encoding gene *sulP*, and methionine aminopeptidase type I-encoding gene *map*. In addition, distinct toxin-antitoxin system-encoding genes were also detected in the two *bla*_OXA-58_-harboring plasmids (Fig. 1A and B), such as *stbD* and *relE* (pAs1069_a) and *brnT* and *brnA* (pAb45063_b). Although *xreE* was found in the large (183-kb) pAb45063_a plasmid (Fig. 1D), no toxin-antitoxin systems were found in the 13-kb pAs1069_b plasmid (Fig. 1C).

## DISCUSSION

The plasmid carrying *bla*_OXA-58_, pAs1069_a, recovered from A. seifertii, shares 99% identity with the plasmid harboring *bla*_OXA-58_ pAb242_25 described in a MDR A. baumannii clinical strain (Ab242) isolated in the city of Rosario, Argentina (4). Interestingly, Ab242 strain was recovered in 1997 (11), 4 years later than the two clonally related OXA-58-producing A. seifertii clinical strains isolated in Brazil (8). Also, Narciso and colleagues described an OXA-58-producing A. seifertii strain (Ac-12.1) recovered in 2012 from a cloaca of a black-necked swan residing in the lakes of the São Paulo Zoo (9). This strain was clonally related to both A. seifertii clinical strains—including the Asp-1069 evaluated in the present study—isolated 19 years earlier in a tertiary hospital located in the city of São Paulo (8, 9). Since the genetic environment surrounding *bla*_OXA-58_ of Ac-12.1 was identical to that of the corresponding gene in the Asp-1069 strain, except for a truncated copy of 3′-IS*Aba3* (8, 9), it reinforces the idea of the capability of rearrangement and the complexity of transposable elements among plasmid-borne *bla*_OXA-58_ genes (3).

Although the OXA-58-producing A. baumannii Acb-45063 strain was included in ST15^IP^, it belongs to same clonal complex (CC15^IP^/CC103^OX^ [Oxford scheme]) as the Ab242 ST104^OX^ A. baumannii strain recovered in Argentina (4). Note that the city of Porto Alegre, where the Acb-45063 strain was recovered, is located in a Brazilian state next to the Argentinian border, where *bla*_OXA-58_ is prevalent and of public health concern (4, 10). However, the plasmids carrying *bla*_OXA-58_ detected in Argentinean and Brazilian A. baumannii strains showed distinct genetic backbones. Although plasmids carrying *bla*_OXA-58_ that belonged to GR8/GR23 were found among distinct Acinetobacter species in South American countries in the 1990s, a distinct genetic backbone surrounding *bla*_OXA-58_ was found in a GR4 plasmid from a A. baumannii Acb-45063 strain recovered in Brazil at 2010. According to Ravasi and colleagues, the presence of IS*Aba825* upstream of *bla*_OXA-58_, as observed in pAb45063_b (ΔIS*Aba3*/IS*Aba825-bla*_OXA-58_-IS*Aba3*), results in a hybrid promoter that overexpresses this CHDL, leading to 16-fold and 8-fold increases in the MICs for imipenem and meropenem, respectively (13). Re27-like sites found in pAb45063_b, but not in pAs1069_a, are short genomic sequences implicated in site specific recombination processes involved in the evolution of plasmids, many of them carrying CHDL-encoding genes (4, 10, 14, 15). These sequences have been identified bordering IS*Aba3*-like elements, allowing the occurrence of multiple recombination processes that promote different arrangements and acquisition of *bla*_OXA-58_ by Acinetobacter species (4, 10–12, 14, 15).

Although it has been previously suggested that the presence of virulence-encoding genes does not guarantee the expression of virulence factors and/or bacterial pathogenicity (16), curiously, our study revealed the presence of distinct virulence factors in all plasmids evaluated, some of which had never been described before in Acinetobacter spp. (13, 15–17). The TonB outer membrane protein is associated with iron uptake, and its expression may be related to the survival of the bacterial cell in the lungs and blood (13, 16, 17). The *spl* gene encodes a septicolysin with cytolytic activity related to the invasion of tissues or cells, while *glmM* codes for a phosphoglucosamine mutase that has been related as a highly sensitive predictor of several clinical outcomes (13, 16, 17). Other three genes found, *ppa*, *sulP*, and *map*, have been associated with bacterial pathogenicity (4). In addition, distinct toxin-antitoxin system-encoding genes found in three of four plasmids evaluated ensure the stability of transferable genetic elements in the bacterial host cell (4, 15, 16).

In conclusion, a complex and dynamic backbones were found surrounding the *bla*_OXA-58_ carried by distinct plasmids from A. seifertii and A. baumannii strains recovered 17 years apart in Brazil. Such data demonstrated that although this CHDL-encoding gene has rarely been reported in Brazil, genetic plasticity has occurred over time, composed of a variety of resistance and virulence markers associated with the stability conferred by toxin-antitoxin systems. These findings accounted in part for the success of efforts that have kept plasmids carrying *bla*_OXA-58_ from escaping nosocomial settings for a long period of time.

## MATERIALS AND METHODS

### Ethical approval.

Ethical approval for this study was obtained from Research Ethics Committee from Federal University of São Paulo—UNIFESP/São Paulo Hospital (process number 5158010817).

### Bacterial isolates.

Two OXA-58-producing Acinetobacter species clinical strains, Asp-1069 and Acb-45063, were selected for this study. The A. seifertii Asp-1069 strain was previously characterized (8) and is considered to be the most ancient Acinetobacter species carrying *bla*_OXA-58_ reported worldwide to date. Asp-1069 was recovered in 1993 from a tracheal aspirate of a patient hospitalized in the city of São Paulo, southeastern Brazilian region (8). The Acb-45063 strain was isolated in 2010 from a blood culture drawn from a patient hospitalized in the city of Porto Alegre, southern Brazilian region. For this study, the Acb-45063 strain was identified at the species level as A. baumannii by sequencing of partial regions of the RNA polymerase β subunit (*rpoB*) gene (18). The CHDL-encoding genes were confirmed by PCR followed by DNA sequencing using specific primers (2, 8, 9). MICs of 12 antimicrobial agents (Sigma-Aldrich, St. Louis, USA) were determined by cation-adjusted broth microdilution and interpreted according to European Committee on Antimicrobial Susceptibility Testing (EUCAST) guidelines (http://www.eucast.org/clinical_breakpoints).

### Multilocus sequence typing (MLST).

MLST analyses of Acb-45063 and Asp-1069 strains were performed by double-stranded DNA sequencing of internal regions of seven housekeeping genes (*cpn60*, *fusA*, *gltA*, *pyrG*, *recA*, *rplB*, and *rpoB*) following the Institute Pasteur scheme. Determination of the sequence type (ST) was performed through the A. baumannii MLST website (http://pubmlst.org/abaumannii/). The relationship between novel STs and existing STs was surveyed using the eBURST program (http://eburst.mlst.net/).

### Plasmid DNA extraction using the alkaline lysis method.

For the calculation of the mean size of plasmids, the plasmid DNA extraction was performed by the alkaline lysis method according to the Birnboim and Doly protocol with a few modifications. One colony per bacterial isolate was inoculated into 3 ml of Trypticase soy broth (TSB) (Oxoid, Basingstoke, United Kingdom) in a tube and incubated at 37°C for 20 to 24 h. Aliquots of 1 ml each were subjected to centrifugation at 12,000 rpm for 3 min, and the pellet was resuspended in 100 μl of solution I (2 mg/ml lysozyme, 2% glucose, 10 mM EDTA, 25 mM Tris-HCl [pH 8.0], 1 mg/ml RNase). After 30 min of incubation in an ice bath, 200 μl of solution II (0.2 N NaOH and 1% SDS) was added. The supernatants were then homogenized by inversion and kept in an ice bath for 7 min. Then, 150 μl of solution III (3 M sodium acetate, pH 4.8) was added to the supernatants and homogenized by inversion and kept in an ice bath for 90 min for the sedimentation of chromosomal DNA. After that, the supernatants were centrifuged at 12,000 rpm for 10 min and transferred to new tubes, and 1 ml of ice-cold ethanol was added. The solution was homogenized by inversion to precipitate the plasmid DNA and incubated at –20°C overnight. The supernatants were centrifuged at 12,000 rpm for 10 min and resuspended in 100 μl of solution IV (100 mM sodium acetate, pH 8.0). Plasmid DNA was precipitated again by the addition of 200 μl of ice-cold ethanol and incubated at –20°C overnight. Finally, a new centrifugation was performed at 12,000 rpm for 10 min. The supernatants were discharged, and pellets were air dried and resuspended in 20 μl of sterile Mili-Q water. The plasmid DNA extractions were stored at –20°C. Electrophoresis was performed on 0.8% agarose (110 V/50 mA) for 2 h and stained with ethidium bromide. The calculation of the estimated plasmid sizes was based on a standard strain with known plasmid sizes running on the same agarose gel using a logarithmic curve.

### Plasmid extraction and whole-plasmid sequencing (WPS).

For WPS, the pool of plasmids was extracted using a QIAPrep Spin MiniPrep extraction kit (Qiagen, Hilden, Germany), concentrated in a Concentrator Plus evaporator (Eppendorf, Hamburg, Germany), and then quantified on a digital Nanovue Plus spectrophotometer (GE Healthcare, Canada). For library preparation, the extractions were quantified again in a Qubit 3.0 Fluorometer (Thermo Fisher Scientific, DE, USA). Libraries were constructed using an Illumina TruSeq Nano DNA LT library preparation kit—set A (Illumina, CA, USA) generating ∼550-bp fragments. WPS analysis was performed in a MiSeq platform (Illumina, CA, USA) (2 × 300 bp) in paired-end mode. The quality and quantification of the libraries were evaluated by quantitative real-time PCR (qRT-PCR).

### Plasmid assembly, automatic annotation, and manual validation.

First, plasmid reads were assembled using Newbler 3.0 (Kartchner, AZ, USA) and Ray 2.3.1 software (Université Laval, QC, Canada). The System for Automated Bacterial Integration of Annotation pipeline (SABIA; available in http://www.sabia.lncc.br) was used for gene prediction and automatic annotation with 90% coverage, 90% similarity, and an E value of <10^−5^. For manual validation, the following platforms were used: NCBI BLAST, UniProt, ISFinder, ResFinder 2.1, Plasmid Finder 1.3, MLSTFinder, and VirulenceFinder 1.5. Creation of the illustration of the circularized plasmids and *in silico* analysis of A. baumannii replicon type group (AbGR) (19) were performed using Snap Gene software 3.3.3 (GSL Biotech LLC, Chicago, USA). BPROM (Softberry Inc, New York, USA) was applied for predicting promoter sequences. The genetic structures surrounding *bla*_OXA-58_ genes were analyzed according to a study previously published by Poirel and Nordmann (20).

### Data accessibility.

The complete nucleotide sequences of pAs1069_a, pAs1069_b, pAb45063_a, and pAb45063_b have been submitted to GenBank under accession numbers MK323040, MK323041, MK323042, and MK323043, respectively.
